# Supplementary material for: Environmental exposures associated with the gut microbiome and resistome of pregnant women and children in Northwest Ecuador
Source: Nat Commun. 2025 Dec 13;17:15. doi: 10.1038/s41467-025-66567-1 (PMC12764814; doi:10.1038/s41467-025-66567-1)
Supplement: Supplementary file 1 — Supplementary Information [file 41467_2025_66567_MOESM1_ESM.pdf]

Environmental exposures associated with the gut microbiome and resistome of pregnant women and children in Northwest Ecuador

Irmarie Cotto[1], Viviana Albán[1±], Ana Durán-Viseras[2±], Kelsey J. Jesser[1], Nicolette A. Zhou[1], Caitlin Hemlock[1], April M. Ballard[3], Christine S. Fagnant-Sperati[1], Gwenyth O. Lee[4,5], Janet K. Hatt[2], Charlotte J. Royer[2,6], Joseph N. S. Eisenberg[7], Gabriel Trueba[8], Konstantinos T. Konstantinidis[2], Karen Levy[1*], Erica R. Fuhrmeister[1,9*] on behalf of the ECoMiD Authorship Group[§]

[1]Department of Environmental and Occupational Health Sciences, University of Washington, Seattle, Washington, USA

[2]School of Civil and Environmental Engineering and School of Biological Sciences, Georgia Institute of Technology, Atlanta, Georgia, USA

[3]Department of Population Health Sciences, Georgia State University, Atlanta, Georgia, USA

[4]Rutgers Global Health Institute, Rutgers University, New Brunswick, New Jersey, USA

[5]Department of Biostatistics and Epidemiology, Rutgers School of Public Health, New Brunswick, New Jersey, USA

[6]Emory School of Medicine, Emory University, Atlanta, Georgia, USA

[7]Department of Epidemiology, University of Michigan, Ann Arbor, Michigan, USA

[8]Instituto de Microbiología, Colegio de Ciencias Biologicas y Ambientales, Universidad San Francisco de Quito, Quito, Ecuador

[9]Department of Civil and Environmental Engineering, University of Washington, Seattle, Washington, USA

[±] Equal contributions

[§]A complete author list for consortium is provided below

*Co-corresponding authors:

Karen Levy, University of Washington, klevyx@uw.edu

Erica R. Fuhrmeister, University of Washington, efuhrm@uw.edu

ECoMiD Authorship Group:
<u>Principal Investigators/Co-Investigators</u>
Karen Levy[1], Joseph N.S. Eisenberg[7], Gwenyth O. Lee[4,5], Gabriel Trueba[8], Benjamin F. Arnold[10], Konstantinos T. Konstantinidis[2], William Cevallos[11]

<u>Field Data Collection Subgroup</u>
Adriana Lupero[8], Mauricio Ayoví[8], Molly K. Miller-Petrie[1]

<u>Data Management Subgroup</u>
Jesse Contreras[7], Jessica Uruchima[7]

<u>Laboratory Analysis Subgroup</u>
- *Lab coordination*: Christine Fagnant-Sperati[1], Gabriela Vasco[12], Stuart Torres[8]
- *Gut microbiome*: Janet Hatt[2], Ana Duran-Viseras[2], Kelsey Jesser[1]

<u>Animal Exposure Subgroup</u>
- *Qualitative & Survey Data*: April Ballard[3], Bethany Caruso[13], Betty Corozo[14]
- *Microbiology*: Kelsey Jesser[1], Viviana Albán[1], Gabriel Trueba[8], Analía Galarza[8]

[10] University of California San Francisco, Proctor Foundation and Department of Opthamology, San Francisco, CA, USA
[11] Universidad Central del Ecuador, Instituto de Biomedicina, Quito, Ecuador
[12] Universidad Central del Ecuador, Facultad de Ciencias Médicas, Carrera de Medicina, Quito, Ecuador
[13] Emory University, Department of Global Health, Atlanta, Georgia, USA
[14] Universidad Técnica Luis Vargas Torres de Esmeraldas, Esmeraldas, Ecuador

Household Animal Presence and Environmental Fecal Contamination

In the study communities along Ecuador's northwestern coast, a wide array of domestic and semi-domestic animals is common in and around household compounds. Households frequently keep poultry (primarily chickens) that are allowed to roam freely throughout yards and communal pathways, depositing feces wherever they forage[1] with 22 of 84 households owning chickens in this study. Dogs and cats, whether owned (46 of 84 households and 20 of 84 households, respectively) or free-roaming strays, move in and out of houses and play areas, further spreading animal feces across domestic spaces. Livestock such as pigs are often penned near the home but sometimes released during the day to scavenge (7 households own in this study), while larger stock, including cattle and horses, graze in nearby fields that children traverse daily (1 household own in this study). Animal feces management practices reported by mothers typically involve rinsing or sweeping droppings into open drainage ditches or into adjacent yards rather than containing or treating them, leading to persistent environmental contamination around homes and communal areas. Even households without animal ownership observed feces tracking into living spaces because roaming animals defecate indiscriminately, highlighting that exposure pathways extend beyond owned-animal contacts to a broader community-level norm of free-range animal husbandry. Additional details on animal exposure in this region are available elsewhere.[1–3]

Metagenomic Results Considering Sequencing Coverage

To ensure our results are robust and not due to differential coverage (or differences in the fraction of diversity that was sequenced), we employed the Nonpareil tool to assess and standardize coverage levels. Nonpareil estimates the redundancy and diversity of metagenomic datasets without reliance on reference databases, providing a robust measure of sequencing coverage.[4,5] We calculated the Nonpareil coverage (Npc) for each metagenome. The Npc values for maternal samples ranged from 0.68 to 0.94, while those for child samples ranged from 0.67 to 0.99. To ensure comparability, we excluded samples where the Npc difference exceeded 0.2 units within each group, based on the metagenome with the highest Npc, resulting in the removal of five maternal and four child samples. We analyzed the impact of exposures in the reduced dataset using the same regression models described in the main text. The results were consistent with our original findings, indicating that sequencing coverage differences did not influence our results. As an additional check, we applied a coverage standardization script based on Nonpareil estimates[6] to normalize ARG abundance data. This approach adjusts for sequencing depth variations, ensuring that observed differences in ARG profiles are not artifacts of sequencing effort. Post-standardization, the ARG outcomes were nearly identical to the initial results, reinforcing that our findings are not due to differences in the diversity that was sequenced.

Animal Feces Culture and Susceptibility Testing

Animal fecal samples (*n=47*) were collected from EcoMid households enrolled in the Animal Exposure (AnEx) sub-study during September 2021 and August-September 2022. AnEx is a mixed-methods study that uses qualitative and quantitative methods to gain a comprehensive understanding of infant's exposure to animals and their feces. Samples were obtained from dogs (*n=19*), chickens (*n=22*), pigs (*n=3*), cows (*n=2*) and ducks (*n=1*). To screen for ESBL-producing bacteria and isolate *E. coli*, ~ 0.1g of stool sample was suspended in 1 mL of 0.85% NaCl solution, vortexed for 1 minute, and centrifuged at 100 x g for 30 seconds; 0.5 mL of supernatant was inoculated in 0.5 mL of non-selective tryptic soy broth and incubated at 37°C overnight. Then, 0.1 mL of enrichment broth was streaked onto selective ChromoSelect ESBL agar and incubated at 37°C overnight. Colonies were classified by manufacturer-specified chromogenic reactions as

presumptive ESBL *E. coli*, ESBL KEC (*Klebsiella*, *Enterobacter* and *Citrobacter*), or ESBL *Pseudomonas/Acinetobacter*. Presumptive ESBL *E. coli* isolates underwent disk-diffusion susceptibility testing: a 0.5 McFarland bacterial solution was inoculated onto Mueller-Hinton agar, commercial antimicrobial-impregnated disks were placed on the agar surface, and plates were incubated at 37°C overnight. Inhibition zones were measured and interpreted as susceptible, intermediate or resistant, according to CLSI M100 Performance Standards.

Table S1: Samples included in this study.

Household ID	Age				
	37w	01w	03m	06m	18m
1001		*	*	*	*
1002	*	*	*	*	*
1003	*	*	*	*	*
1004	*	*	*	*	*
1101	*	*	*	*	*
1102	*	*	*	*	*
1103	*	*	*	*	*
1104	*	*	*	*	*
1106	*	*	*	*	*
2002	*	*	*	*	*
2003	*	*	*	*	*
2202	*		*	*	
2204	*	*	*	*	*
2301	*	*	*	*	*
2302	*	*	*	*	*
3001	*	*	*	*	*
3101	*	*	*	*	*
3201	*	*	*	*	*
7001	*	*	*	*	*
7001	*	*	*	*	
1007	*	*			*
1107	*	*			*
2009	*	*			*
2101	*	*			*
2205	*	*			*
2206	*	*			*
2306	*	*			*
3004	*	*			*
3006	*	*			*
3102	*	*			*
6001	*	*			*
6006	*	*			*
8001	*	*			
8002	*	*			
8003	*	*			
2001	*	*			
2004	*	*			
2008	*	*			
2102	*	*			
2103	*	*			
2201	*	*			
2304	*	*			
2305	*	*			
2308	*	*			

2311	*	*	
4002	*	*	
1005	*	*	
1006	*	*	
1011	*	*	
1012	*	*	
3005	*	*	
3103	*	*	
6002	*	*	
6004	*	*	
2213			*
3007			*
3015			*
3108			*
3110			*
3112			*
3115			*
3012			*
3107			*
9009			*
5001			*
9006			*
2317			*
9005			*
1116			*
2119			*
7003			*
2219			*
3002			*
2320			*
3011			*
9003			*
9001			*
2012			*
2322			*
4006			*
2312			*
6009			*
9002			*
7011			*

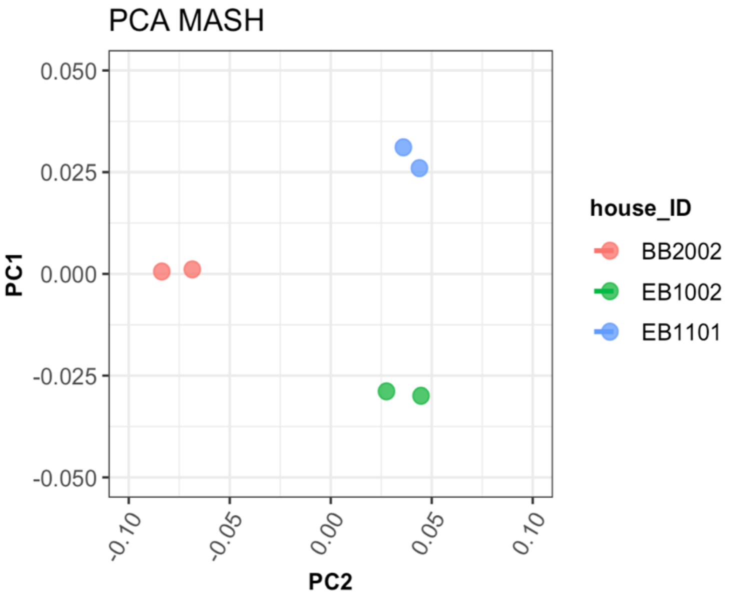

Figure S1: PCA of MASH distance of three 6-month samples sequenced at both Georgia Tech and the University of Washington.

Table S2: Linear model results for the microbial outcomes with animal exposure, DW & San, and piped water availability in mothers.

		**Unadjusted**		**Adjusted**		
	**Exposure**	**β Coefficient (95% CI)**	**p-value**	**β Coefficient (95% CI)**	**p-value**	**N**
**No. of unique clinically relevant ARGs**	**Animal Exposure**					
	High	Reference		Reference		4
	Medium	-4.20 (-10.52, 2.12)	0.20	-5.31 (-11.40, 0.78)	0.09	10
	Low	-5.36 (-11.41, 0.70)	0.09	-5.67 (-12.83, -0.51)	0.04	14
	Zero	-0.30 (-6.05, 5.45)	0.92	-3.24 (-9.06, 2.57)	0.28	25
	**Sanitation & DW**					
	Other	Reference		Reference		21
	CS&PW	-4.26 (-7.35, -1.17)	0.01	-4.16 (-7.53, -0.78)	0.02	30
	**Piped Water**					
	Not piped	Reference		Reference		14
	0-6 days	-3.26 (-6.96, 0.43)	0.09	-2.23 (-6.57, 2.09)	0.49	21
	7 days	-5.63 (-9.45, -1.82)	0.01	-3.47 (-8.20, 1.25)	0.16	18
**Nonpareil Sequence Diversity**	**Animal Exposure**					
	High	Reference		Reference		4
	Medium	-0.94 (-1.62, -0.27)	0.01	-1.07 (-1.70, -0.43)	0.002	10
	Low	-1.18 (-1.83, -0.54)	0.001	-1.58 (-2.23, -0.95)	1.7e-05	14
	Zero	-0.93 (-1.54, -0.31)	0.005	-1.35 (-1.96, -0.75)	7.8e-05	25
	**Sanitation & DW**					
	Other	Reference		Reference		21
	CS&PW	-0.08 (-0.45, 0.28)	0.66	0.01 (-0.45, 0.43)	0.97	30
	**Piped Water**					
	Not piped	Reference		Reference		14
	0-6 days	0.06 (-0.36, 0.49)	0.77	0.29 (-0.22, 0.81)	0.27	21
	7 days	-0.32 (-0.75, 0.12)	0.16	-0.27 (-0.84, 0.29)	0.34	18
**Clinically Relevant**	**Animal Exposure**					
	High	Reference		Reference		4
	Medium	0.01 (-0.06, 0.08)	0.78	0.00 (-0.07, 0.07)	0.99	10

**ARG Abundance**	Low	-0.03 (-0.10, 0.03)	0.33	-0.04 (-0.11, 0.03)	0.26	14
	Zero	0.00 (-0.06, 0.06)	0.97	-0.02 (-0.08, 0.05)	0.59	25
	**Sanitation & DW**					
	Other	Reference		Reference		21
	CS&PW	-0.03 (-0.06, 0.01)	0.14	-0.02 (-0.06, 0.02)	0.31	30
	**Piped Water**					
	Not piped	Reference		Reference		14
	0-6 days	-0.06 (-0.09, -0.02)	0.004	-0.06 (-0.10, -0.01)	0.02	21
	7 days	-0.06 (-0.09, -0.02)	0.005	-0.04 (-0.09, 0.01)	0.09	18
**% *E. coli* Relative Abundance**	**Animal Exposure**					
	High	Reference		Reference		4
	Medium	-0.24 (-0.80, 0.33)	0.41	-0.34 (-0.90, 0.22)	0.24	9
	Low	-0.24 (-0.77, 0.29)	0.38	-0.46 (-1.02, 0.10)	0.12	14
	Zero	0.12 (-0.39, 0.62)	0.65	0.04 (-0.57, 0.49)	0.88	25
	**Sanitation & DW**					
	Other	Reference		Reference		21
	CS&PW	-0.30 (-0.57, -0.03)	0.04	-0.26 (-0.58, 0.06)	0.12	29
	**Piped Water**					
	Not piped	Reference		Reference		13
	0-6 days	-0.07 (-0.41, 0.27)	0.70	0.22 (-0.19, 0.64)	0.29	21
	7 days	-0.29 (-0.64, 0.06)	0.11	-0.15 (-0.59, 0.29)	0.50	18
**% *K. pneumoniae* Relative Abundance**	**Animal Exposure**					
	High	Reference		Reference		4
	Medium	0.00 (-0.64, 0.63)	0.99	-0.04 (-0.74, 0.65)	0.90	10
	Low	0.00 (-0.61, 0.61)	0.99	-0.08 (-0.78, 0.62)	0.82	14
	Zero	0.30 (-0.28, 0.88)	0.32	0.20 (-0.47, 0.86)	0.56	25
	**Sanitation & DW**					
	Other	Reference		Reference		21
	CS&PW	-0.04 (-0.36, 0.28)	0.81	0.05 (-0.35, 0.44)	0.82	30
	**Piped Water**					
	Not piped	Reference		Reference		14
	0-6 days	-0.24 (-0.62, 0.14)	0.22	-0.50 (-0.95, 0.04)	0.04	21
	7 days	-0.15 (-0.54, 0.24)	0.44	-0.10 (-0.59, 0.40)	0.70	18

Table S3: Linear model results for the microbiological outcomes with animal exposure, DW & San and piped water availability in children.

		**Unadjusted**		**Adjusted**		
	**Exposure**	**β Coefficient (95% CI)**	**p-value**	**β Coefficient (95% CI)**	**p-value**	**N**
**No. of unique clinically relevant ARGs**	**Animal Exposure**					
	High	Reference		Reference		29
	Medium	7.87 (0.00, 15.73)	0.05	5.47 (-2.47, 13.41)	0.18	31
	Low	11.61 (3.10, 20.12)	0.01	7.92 (-0.51, 16.34)	0.07	32
	Zero	5.44 (0.12, 10.77)	0.05	2.00 (-4.30,8.28)	0.53	58
	**Sanitation & DW**					
	Other	Reference		Reference		47
	CS&PW	-0.45 (-5.99, 5.09)	0.87	-5.45 (-11.12, 0.21)	0.06	103
	**Piped Water**					
	Not piped	Reference		Reference		32
	0-6 days	5.84 (0.15, 11.53)	0.04	4.82 (-1.93, 11.56)	0.16	28
	7 days	3.02 (-3.51, 9.55)	0.36	1.12 (-7.46, 9.70)	0.80	19
	**Animal Exposure**					
	High	Reference		Reference		29

	Medium	-0.33 (-0.83, 0.18)	0.21	0.10 (-0.33, 0.53)	0.65	31
	Low	-0.63 (-1.16, -0.10)	0.02	0.21 (-0.28, 0.70)	0.40	32
	Zero	-0.74 (-1.26, -0.22)	0.005	0.04 (-0.40, 0.49)	0.84	58
	**Sanitation & DW**					
**Nonpareil Sequence Diversity**	Other	Reference		Reference		47
	CS&PW	-0.42 (-0.71, -0.12)	0.005	-0.07 (-0.33, 0.19)	0.61	103
	**Piped Water**					
	Not piped	Reference		Reference		32
	0-6 days	-0.15 (-0.63, 0.33)	0.55	-0.30 (-0.71, 0.11)	0.22	28
	7 days	-0.56 (-1.10, -0.02)	0.04	-0.63 (-1.11, -0.15)	0.01	19
**Clinically Relevant ARG Abundance**	**Animal Exposure**					
	High	Reference		Reference		29
	Medium	0.12 (-0.03, 0.26)	0.11	0.10 (-0.03, 0.23)	0.14	31
	Low	0.09 (-0.02, 0.21)	0.12	0.05 (-0.08, 0.18)	0.42	32
	Zero	0.17 (0.03, 0.31)	0.02	0.11 (-0.06, 0.27)	0.20	58
	**Sanitation & DW**					
	Other	Reference		Reference		47
	CS&PW	-0.07 (-0.16, 0.03)	0.18	-0.10 (-0.22, 0.03)	0.12	103
	**Piped Water**					
	Not piped	Reference		Reference		32
	0-6 days	0.16 (-0.01, 0.33)	0.06	0.12 (-0.03, 0.26)	0.11	28
	7 days	0.05 (-0.03, 0.14)	0.23	0.03 (-0.06, 0.13)	0.49	19
**% *E. coli* Relative Abundance**	**Animal Exposure**					
	High	Reference		Reference		29
	Medium	3.37 (-0.81, 7.56)	0.11	2.68 (-1.14, 6.51)	0.17	31
	Low	0.17 (-2.51, 2.85)	0.90	-1.59 (-4.42, 1.24)	0.27	32
	Zero	3.06 (-0.01, 6.13)	0.05	1.05 (-2.34, 4.44)	0.54	58
	**Sanitation & DW**					
	Other	Reference		Reference		47
	CS&PW	-2.10 (-4.56, 0.36)	0.09	-3.21 (-6.55, -0.12)	0.06	103
	**Piped Water**					
	Not piped	Reference		Reference		32
	0-6 days	2.46 (-0.17, 5.09)	0.07	1.31 (-1.21, 3.83)	0.31	28
	7 days	1.27 (-0.70, 3.24)	0.21	0.58 (-1.46, 2.62)	0.57	19
**% *K. pneumoniae* Relative Abundance**	**Animal Exposure**					
	High	Reference		Reference		29
	Medium	0.59 (-0.77, 1.95)	0.39	-0.20 (-2.37, 1.97)	0.86	31
	Low	3.03 (-0.74, 6.8)	0.12	1.77 (-1.17, 4.72)	0.24	32
	Zero	2.42 (0.03, 4.82)	0.05	0.81 (-1.90, 3.53)	0.56	58
	**Sanitation & DW**					
	Other	Reference		Reference		47
	CS&PW	3.03 (-0.16, 6.21)	0.06	2.27 (-1.16, 5.73)	0.19	103
	**Piped Water**					
	Not piped	Reference		Reference		32
	0-6 days	1.17 (-0.18, 2.52)	0.09	1.40 (-0.45, 3.26)	0.14	28
	7 days	-0.96 (-2.44, 0.53)	0.21	-1.45 (-4.03, 1.12)	0.27	19

Table S4: Linear model results for animal exposure in subgroups (mothers).

	Sub group	Animal Exposure	Unadjusted β Coefficient (95% CI)	Unadjusted p-value	Adjusted β Coefficient (95% CI)	Adjusted p-value	Diff. Models p-value	N
**No. of unique clinically relevant ARGs**	SC & PW	High	Reference		Reference		0.11	2
		Medium	-1.83 (-8.85, 5.19)	0.62	-4.55 (-13.04, 3.93)	0.31		6
		Low	-5.67 (-12.69,1.35)	0.13	-8.92 (-16.43, -1.42)	0.04		6
		Zero	2.14 (-4.75, 9.04)	0.55	-3.38 (-11.71, 4.96)	0.44		7
	Other	High	Reference		Reference			2
		Medium	-6.00 (-15.90, 3.92)	0.25	-11.49 (-21.77, -1.21)	0.04		4
		Low	-6.00 (-15.10, 3.05)	0.21	-8.92 (-18.66, 0.82)	0.09		8
		Zero	-3.31 (-11.90, 5.28)	0.46	-6.25 (-14.91, 2.42)	0.17		16
**Nonpareil Sequence Diversity**	SC & PW	High	Reference		Reference		0.41	2
		Medium	-1.05 (-2.12, 0.01)	0.07	-1.42 (-3.00, 0.15)	0.10		6
		Low	-1.75 (-2.82, -0.69)	0.005	-2.25 (-3.64, -0.86)	0.01		6
		Zero	-1.13 (-2.18, -0.09)	0.05	-1.78 (-3.32, -0.23)	0.05		7
	Other	High	Reference		Reference			2
		Medium	-0.89 (-1.79, 0.02)	0.07	-1.01 (-1.94, -0.09)	0.04		4
		Low	-0.70 (-1.53, 0.12)	0.11	-1.99 (-1.8, -0.24)	0.02		8
		Zero	-0.68 (-1.46, 0.10)	0.10	-1.94 (-1.75, -0.38)	0.01		16
**Clinically Relevant ARG Abundance**	SC & PW	High	Reference		Reference		0.33	2
		Medium	-0.02 (-0.07, 0.03)	0.38	-0.00 (-0.06, 0.05)	0.86		6
		Low	-0.04 (-0.08, 0.01)	0.16	-0.02 (-0.07, 0.03)	0.39		6
		Zero	0.00 (-0.04, 0.05)	0.87	0.02 (-0.04, 0.07)	0.60		7
	Other	High	Reference		Reference			2
		Medium	0.06 (-0.06, 0.18)	0.34	0.03 (-0.11, 0.17)	0.67		4
		Low	-0.03 (-0.14, 0.08)	0.57	-0.05 (-0.18, 0.09)	0.51		8
		Zero	0 (-0.11, 0.10)	0.95	-0.01 (-0.13, 0.11)	0.90		16
**% *E. coli* Relative Abundance**	SC & PW	High	Reference		Reference		0.68	2
		Medium	-0.32 (-0.63, -0.02)	0.06	-0.52 (-0.92, -0.11)	0.03		6
		Low	-0.43 (-0.74, -0.13)	0.01	-0.62 (-0.99, -0.26)	0.01		6
		Zero	-0.01 (-0.31, 0.30)	0.97	-0.12 (-0.52, 0.28)	0.56		7
	Other	High	Reference		Reference			2
		Medium	-0.07 (-1.16, 1.02)	0.90	-0.86 (-2.05, 0.32)	0.17		3
		Low	-0.09 (-1.03, 0.85)	0.85	-0.91 (-1.99, 0.17)	0.12		8
		Zero	0.22 (-0.68, 1.11)	0.64	0.47 (-1.44, 0.49)	0.35		16
**% *K. pneumoniae* Relative Abundance**	SC & PW	High	Reference		Reference		0.99	2
		Medium	-0.02 (-0.54, 0.51)	0.95	-0.16 (-0.80, 0.48)	0.63		6
		Low	-0.04 (-0.57, 0.49)	0.88	-0.07 (-0.64, 0.49)	0.81		6
		Zero	0.32 (-0.20, 0.84)	0.24	0.42 (-0.21, 1.05)	0.21		7
	Other	High	Reference		Reference			2
		Medium	0 (-1.20, 1.20)	1	0.09 (-1.37, 1.55)	0.91		4
		Low	0.03 (-1.06, 1.12)	0.95	0.27 (-1.12, 1.65)	0.71		8
		Zero	0.32 (-0.71, 1.36)	0.55	0.53 (-0.70, 1.77)	0.41		16

Table S5: Linear model results for animal exposure in subgroups (children).

	Sub group	Animal Exposure	Unadjusted β Coefficient (95% CI)	Unadjusted p-value	Adjusted β Coefficient (95% CI)	Adjusted p-value	Diff. Models p-value	N
**No. of unique clinically relevant ARGs**	SC & PW	High	Reference		Reference		0.005	3
		Medium	23.51 (13.38, 33.60)	5.5e-06	16.72 (-9.56, 42.99)	0.21		10
		Low	18.08 (7.28, 28.90)	0.001	10.42 (-14.65, 35.49)	0.41		15
		Zero	9.65 (2.11, 17.20)	0.01	15.50 (-8.06, 39.06)	0.20		18
	Other	High	Reference		Reference			26
		Medium	2.99 (-5.24, 11.20)	0.48	1.62 (-6.78, 10.01)	0.71		21
		Low	9.32 (-3.14, 21.80)	0.14	6.91 (-5.57, 19.39)	0.28		16
		Zero	6.23 (0.05, 12.40)	0.05	3.71 (-4.04, 11.46)	0.35		38
**Nonpareil Sequence Diversity**	SC & PW	High	Reference		Reference		0.003	3
		Medium	0.85 (-0.10, 1.80)	0.08	1.23 (0.63, 1.82)	6.7e-05		10
		Low	0.82 (-0.09, 1.72)	0.08	1.16 (0.62, 1.69)	5.3e-05		15
		Zero	0.72 (-0.15, 1.58)	0.10	0.81 (0.34, 1.27)	0.001		18
	Other	High	Reference		Reference			26
		Medium	-0.31 (-0.84, 0.22)	0.25	-0.12 (-0.65, 0.41)	0.66		21
		Low	-0.76 (-1.41, -0.12)	0.02	0.22 (-0.31, 0.75)	0.42		16
		Zero	-0.91 (-1.48, -0.34)	0.002	0.10 (-0.50, 0.70)	0.74		38
**Clinically Relevant ARG Abundance**	SC & PW	High	Reference		Reference		0.36	3
		Medium	0.09 (-0.04, 0.22)	0.19	0.10 (-0.02, 0.22)	0.10		10
		Low	0.07 (-0.09, 0.23)	0.41	0.05 (-0.08, 0.19)	0.47		15
		Zero	0.06 (-0.05, 0.16)	0.28	0.15 (0.04, 0.25)	0.006		18
	Other	High	Reference		Reference			26
		Medium	0.13 (-0.06, 0.32)	0.16	0.18 (0.00, 0.36)	0.05		21
		Low	0.13 (-0.03, 0.28)	0.10	0.07 (-0.10, 0.24)	0.40		16
		Zero	0.24 (0.04, 0.44)	0.02	0.18 (-0.07, 0.43)	0.16		38
**% *E. coli* Relative Abundance**	SC & PW	High	Reference		Reference		0.04	3
		Medium	6.90 (2.97, 10.82)	0.0006	7.00 (2.90, 11.09)	0.001		10
		Low	1.37 (-0.51, 3.26)	0.15	1.06 (-1.93, 4.05)	0.49		15
		Zero	2.79 (0.13, 5.46)	0.04	2.92 (-0.71, 6.55)	0.11		18
	Other	High	Reference		Reference			26
		Medium	2.73 (-2.83, 8.28)	0.34	2.90 (-1.80, 7.59)	0.23		21
		Low	1.68 (-1.56, 4.93)	0.31	-2.22 (-6.37, 1.92)	0.29		16
		Zero	4.25 (0.26, 8.24)	0.04	0.69 (-4.49, 5.86)	0.80		38
**% *K. pneumoniae* Relative Abundance**	SC & PW	High	Reference		Reference		0.12	3
		Medium	2.33 (0.12, 4.54)	0.04	1.55 (-8.39, 11.49)	0.76		10
		Low	5.62 (-0.58, 11.82)	0.08	6.63 (-2.52, 15.78)	0.16		15
		Zero	4.37 (0.00, 8.74)	0.05	8.51 (-0.94, 17.96)	0.08		18
	Other	High	Reference		Reference			26
		Medium	-1.03 (-2.13, 0.07)	0.07	-3.12 (-5.36, -0.88)	0.01		21
		Low	0.46 (-2.33, 3.25)	0.75	-1.43 (-3.24, 0.37)	0.12		16
		Zero	1.44 (-0.81, 3.7)	0.21	-0.71 (-2.62, 1.20)	0.47		38

Table S6: Linear model results for the microbial outcomes with animal exposure, DW & San, and piped water availability in female children.

	Exposure	Unadjusted		Adjusted		N
		β Coefficient (95% CI)	p-value	β Coefficient (95% CI)	p-value	
**No. of unique clinically relevant ARGs**	**Animal Exposure**					
	High	Reference		Reference		15
	Medium	1.33 (-4.92, 7.58)	0.68	6.77 (-2.97, 16.50)	0.17	12
	Low	1.21 (-4.72, 7.14)	0.69	12.97 (4.41, 21.54)	0.003	17
	Zero	4.00 (-1.28, 9.27)	0.14	15.07 (7.47, 22.68)	0.0001	26
	**Sanitation & DW**					
	Other	Reference		Reference		48
	CS&PW	2.48 (-3.03, 7.99)	0.38	5.96 (1.41, 10.50)	0.01	22
	**Piped Water**					
	Not piped	Reference		Reference		17
	0-6 days	4.25 (-5.44, 13.94)	0.39	8.91 (-0.22, 18.03)	0.06	13
	7 days	1.47 (-8.12, 11.05)	0.76	0.72 (-6.19, 7.62)	0.84	7
**Nonpareil Sequence Diversity**	**Animal Exposure**					
	High	Reference		Reference		15
	Medium	-0.43 (-1.28, 0.41)	0.31	-0.04 (-0.77, 0.69)	0.92	12
	Low	-0.58 (-1.35, 0.18)	0.13	0.12 (-0.50, 0.75)	0.70	17
	Zero	-0.59 (-1.41, 0.24)	0.16	-0.10 (-0.71, 0.51)	0.75	26
	**Sanitation & DW**					
	Other	Reference		Reference		48
	CS&PW	-0.43 (-0.9, 0.04)	0.07	-0.08 (-0.30, 0.14)	0.49	22
	**Piped Water**					
	Not piped	Reference		Reference		17
	0-6 days	-0.60 (-1.12, -0.08)	0.02	-0.31 (-0.81, 0.19)	0.23	13
	7 days	-1.23 (-2.01, -0.45)	0.002	-0.84 (-1.37, -0.30)	0.002	7
**Clinically Relevant ARG Abundance**	**Animal Exposure**					
	High	Reference		Reference		15
	Medium	0.09 (-0.19, 0.37)	0.53	0.11 (-0.16, 0.38)	0.42	12
	Low	0.04 (-0.13, 0.21)	0.68	0.17 (-0.07, 0.40)	0.16	17
	Zero	0.19 (-0.1, 0.49)	0.20	0.25 (-0.06, 0.56)	0.11	26
	**Sanitation & DW**					
	Other	Reference		Reference		48
	CS&PW	-0.17 (-0.34, 0.01)	0.06	-0.18 (-0.39, 0.02)	0.08	22
	**Piped Water**					
	Not piped	Reference		Reference		17
	0-6 days	0.30 (-0.02, 0.62)	0.06	0.26 (0.01, 0.52)	0.04	13
	7 days	0.03 (-0.04, 0.09)	0.42	0.03 (-0.10, 0.17)	0.61	7
**% *E. coli* Relative Abundance**	**Animal Exposure**					
	High	Reference		Reference		15
	Medium	1.77 (-7.14, 10.69)	0.70	1.48 (-6.25, 9.21)	0.71	12
	Low	-2.06 (-5.85, 1.72)	0.29	-0.98 (-5.23, 3.28)	0.65	17
	Zero	2.25 (-3.11, 7.6)	0.41	2.13 (-3.56, 7.82)	0.46	26
	**Sanitation & DW**					
	Other	Reference		Reference		48
	CS&PW	-4.06 (-8.14, 0.03)	0.05	-4.61 (-9.22, 0.01)	0.05	22
	**Piped Water**					
	Not piped	Reference		Reference		17
	0-6 days	6.35 (1.41, 11.29)	0.01	5.67 (1.40, 9.94)	0.01	13
	7 days	0.97 (-0.87, 2.80)	0.30	1.38 (-0.86, 3.63)	0.23	7
**% *K. pneumonia***	**Animal Exposure**					
	High	Reference		Reference		15

***e* Relative Abundance**	Medium	-0.01 (-2.00, 1.98)	0.99	-1.58 (-4.00, 0.85)	0.20	12
	Low	0.49 (-0.99, 1.97)	0.52	-0.83 (-2.83, 1.18)	0.42	17
	Zero	2.06 (-0.53, 4.64)	0.12	0.63 (-1.15, 2.40)	0.49	26
	**Sanitation & DW**					
	Other	Reference		Reference		48
	CS&PW	3.34 (1.02, 5.66)	0.005	3.27 (1.22, 5.32)	0.002	22
	**Piped Water**					
	Not piped	Reference		Reference		17
	0-6 days	0.58 (-0.33, 1.48)	0.21	0.58 (-0.09, 1.24)	0.09	13
	7 days	0.13 (-0.18, 0.44)	0.41	0.13 (-0.37, 0.63)	0.60	7

Table S7: Linear model results for the microbial outcomes with animal exposure, DW & San, and piped water availability in male children.

		Unadjusted		Adjusted		
	**Exposure**	**β Coefficient (95% CI)**	**p-value**	**β Coefficient (95% CI)**	**p-value**	**N**
**No. of unique clinically relevant ARGs**	**Animal Exposure**					
	High	Reference		Reference		14
	Medium	13.30 (2.41, 24.18)	0.02	13.42 (0.91, 25.93)	0.04	19
	Low	25.21 (9.99, 40.42)	0.001	24.87 (9.98, 39.76)	0.001	15
	Zero	6.80 (-2.42, 16.03)	0.15	4.99 (-9.05, 19.03)	0.49	32
	**Sanitation & DW**					
	Other	Reference		Reference		55
	CS&PW	1.17 (-7.13, 9.48)	0.78	-10.25 (-17.04, -3.45)	0.003	25
	**Piped Water**					
	Not piped	Reference		Reference		15
	0-6 days	7.16 (-10.43, 24.75)	0.42	4.06 (-12.19, 20.31)	0.62	15
	7 days	0.72 (-18.95, 20.39)	0.94	-3.59 (-13.93, 6.75)	0.50	12
**Nonpareil Sequence Diversity**	**Animal Exposure**					
	High	Reference		Reference		14
	Medium	-0.30 (-0.90, 0.31)	0.34	0.27 (-0.14, 0.68)	0.20	19
	Low	-0.68 (-1.40, 0.04)	0.07	0.47 (-0.17, 1.10)	0.15	15
	Zero	-0.88 (-1.52, -0.24)	0.01	0.21 (-0.27, 0.69)	0.39	32
	**Sanitation & DW**					
	Other	Reference		Reference		55
	CS&PW	-0.43 (-0.80, -0.06)	0.02	-0.05 (-0.44, 0.35)	0.81	25
	**Piped Water**					
	Not piped	Reference		Reference		15
	0-6 days	0.17 (-0.46, 0.81)	0.59	0.23 (-0.23, 0.69)	0.32	15
	7 days	-0.17 (-0.77, 0.43)	0.58	-0.31 (-0.79, 0.17)	0.21	12
**Clinically Relevant ARG Abundance**	**Animal Exposure**					
	High	Reference		Reference		14
	Medium	0.16 (0.03, 0.29)	0.02	0.09 (-0.01, 0.19)	0.07	19
	Low	0.16 (0.01, 0.30)	0.04	0.09 (-0.04, 0.22)	0.19	15
	Zero	0.17 (0.10, 0.23)	3.8e-07	0.03 (-0.08, 0.14)	0.57	32
	**Sanitation & DW**					
	Other	Reference		Reference		55
	CS&PW	0.04 (-0.06, 0.14)	0.46	-0.07 (-0.18, 0.03)	0.17	25
	**Piped Water**					
	Not piped	Reference		Reference		15
	0-6 days	0.01 (-0.08, 0.10)	0.86	0.09 (-0.06, 0.25)	0.23	15
	7 days	0.07 (-0.03, 0.17)	0.15	-0.07 (-0.19, 0.05)	0.25	12
	**Animal Exposure**					

**% *E. coli* Relative Abundance**	High	Reference		Reference		14
	Medium	5.20 (2.08, 8.31)	0.001	4.27 (0.70, 7.85)	0.02	19
	Low	2.43 (-0.50, 5.37)	0.10	1.54 (-2.55, 5.64)	0.46	15
	Zero	4.21 (1.46, 6.95)	0.003	1.06 (-2.87, 4.98)	0.60	32
	**Sanitation & DW**					
	Other	Reference		Reference		55
	CS&PW	-0.34 (-3.21, 2.53)	0.82	-3.65 (-7.67, 0.36)	0.07	25
	**Piped Water**					
	Not piped	Reference		Reference		15
	0-6 days	0.29 (-2.15, 2.74)	0.82	-1.31 (-3.91, 1.29)	0.32	15
	7 days	1.74 (-1.20, 4.68)	0.25	-0.63 (-2.79, 1.54)	0.57	12
**% *K. pneumoniae* Relative Abundance**	**Animal Exposure**					
	High	Reference		Reference		14
	Medium	0.86 (-0.90, 2.62)	0.34	1.56 (-2.38, 5.49)	0.44	19
	Low	6.03 (-2.18, 14.23)	0.15	6.61 (0.08, 13.13)	0.05	15
	Zero	3.04 (-1.71, 7.79)	0.21	1.50 (-4.43, 7.43)	0.62	32
	**Sanitation & DW**					
	Other	Reference		Reference		55
	CS&PW	3.10 (-1.88, 8.08)	0.22	0.83 (-4.37, 6.03)	0.75	25
	**Piped Water**					
	Not piped	Reference		Reference		15
	0-6 days	1.60 (-1.68, 4.88)	0.34	4.30 (-1.53, 10.12)	0.15	15
	7 days	-1.41 (-3.39, 0.58)	0.17	-3.25 (-6.70, 0.20)	0.06	12

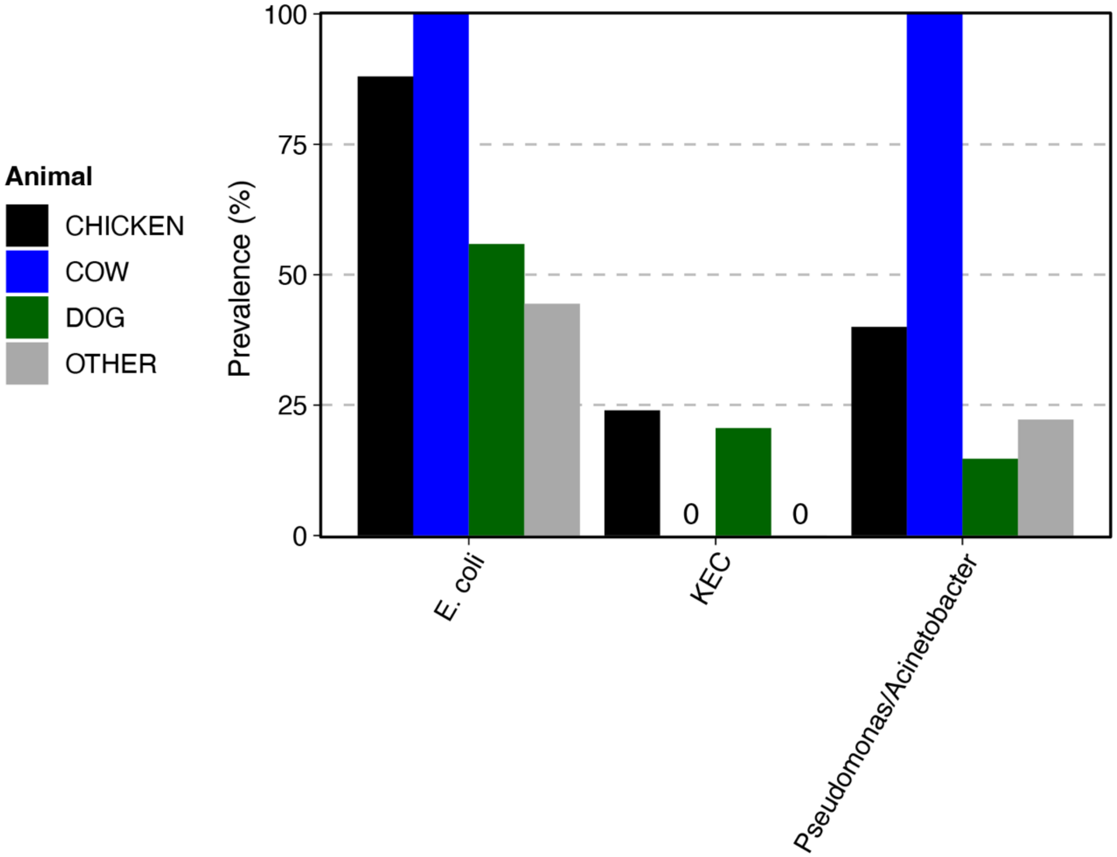

Figure S2: ESBL-producing bacteria prevalence by animal type: dogs (*n=19*), chickens (*n=22*), cows (*n*=2) and other (ducks *n=1*, pigs *n=3*). KEC= *Klebsiella*, *Enterobacter*, *Citrobacter.*

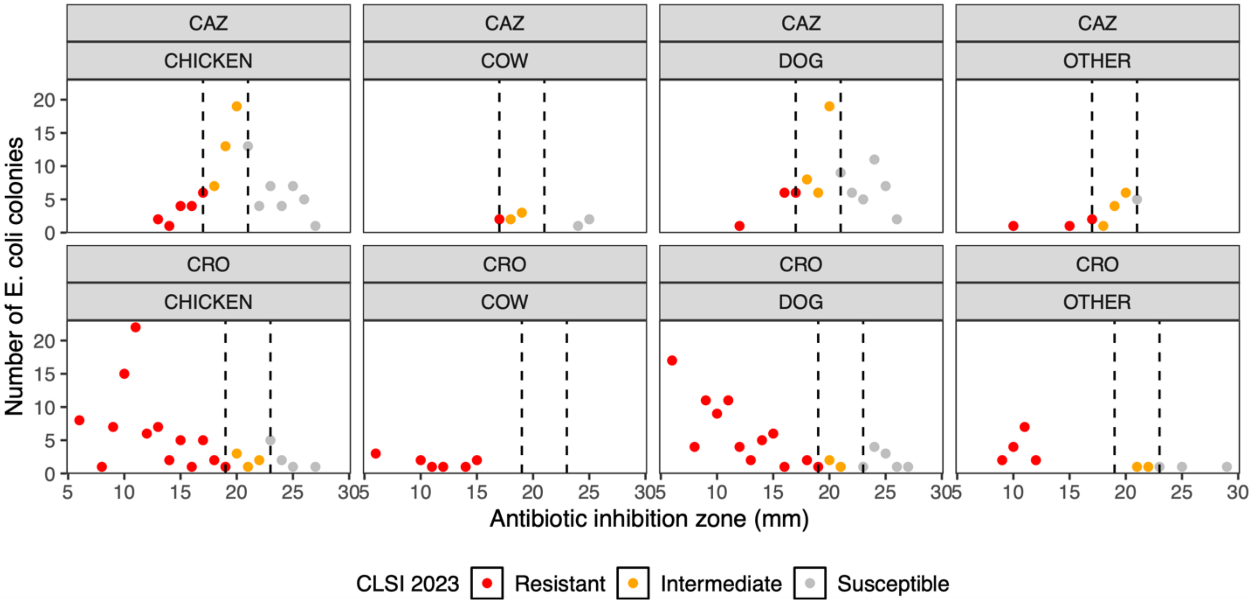

Figure S3: Antibiotics susceptibility profiles of ESBL *E. coli* isolates. CAZ=ceftazidime, CRO=ceftriaxone.

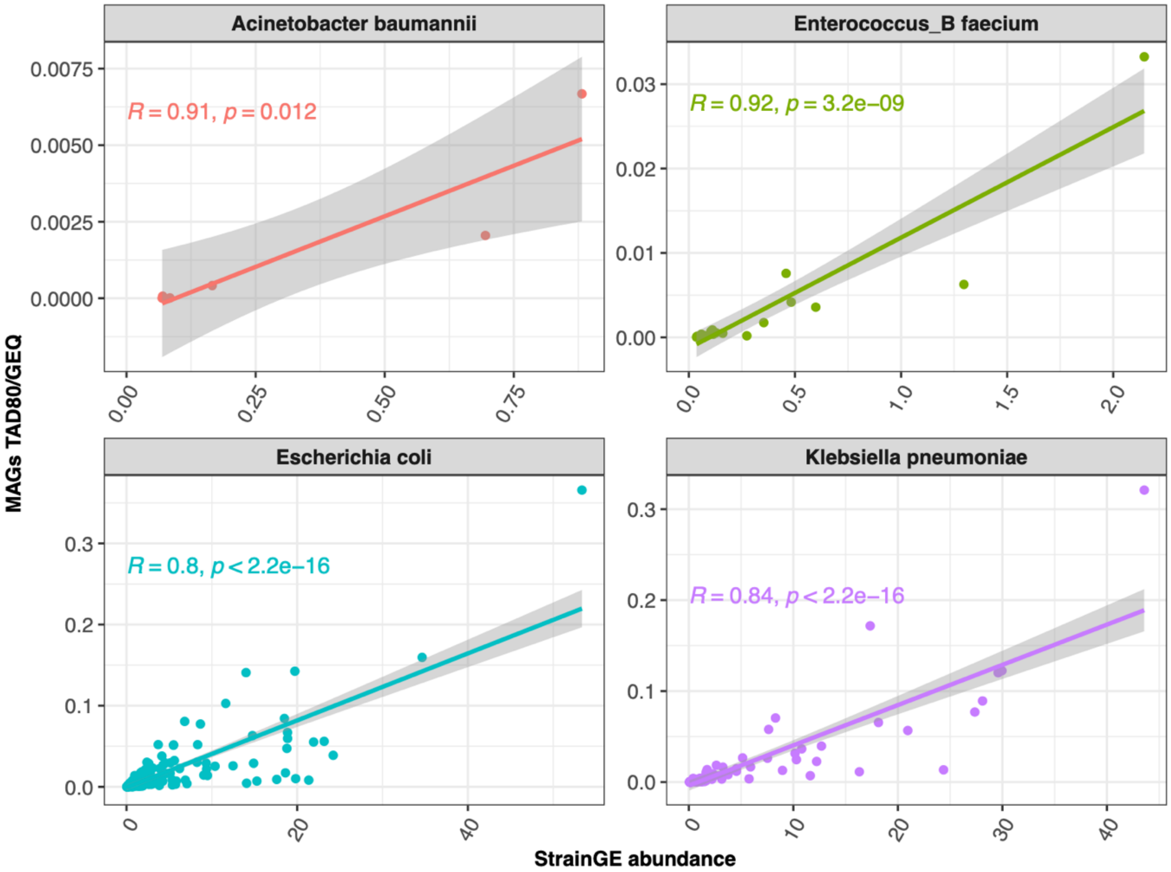

Figure S4: Comparison of ESKAPEE pathogen relative abundances estimated from MAGs and StrainGE.

References

1. Ballard, A. M. *et al.* Multilevel factors drive child exposure to enteric pathogens in animal feces: A qualitative study in northwestern coastal Ecuador. *PLOS Global Public Health* **4**, e0003604 (2024).
2. Ballard, A. M. *et al.* The development and validation of a survey to measure fecal-oral child exposure to zoonotic enteropathogens: The FECEZ Enteropathogens Index. *PLOS Global Public Health* **4**, e0002690 (2024).
3. Jesser, K. J. *et al.* Environmental Exposures Associated with Enteropathogen Infection in Six-Month-Old Children Enrolled in the ECoMiD Cohort along a Rural–Urban Gradient in Northern Ecuador. *Environ Sci Technol* **59**, 103-118 (2024).
4. Rodriguez-R, L. M., Gunturu, S., Tiedje, J. M., Cole, J. R. & Konstantinidis, K. T. Nonpareil 3: Fast Estimation of Metagenomic Coverage and Sequence Diversity. *mSystems* **3**, (2018).
5. Rodriguez-R, L. M. & Konstantinidis, K. T. Nonpareil: a redundancy-based approach to assess the level of coverage in metagenomic datasets. *Bioinformatics* **30**, 629–635 (2014).
6. Riquelme, B. A., Rodriguez-R, L. M. & Konstantinidis, K. T. Differences in metagenome coverage may confound abundance-based and diversity conclusions and how to deal with them. *ISME Communications* **5**, ycaf140 (2025).
