# Supplementary material for: Environmental exposures associated with the gut microbiome and resistome of pregnant women and children in Northwest Ecuador
Source: Nat Commun. 2025 Dec 13;17:15. doi: 10.1038/s41467-025-66567-1 (PMC12764814; doi:10.1038/s41467-025-66567-1)
Supplement: Supplementary file 2 — Description of Additional Supplementary Files [file 41467_2025_66567_MOESM2_ESM.pdf]

Description of Additional Supplementary Files

File Name: Supplementary Data 1

Description: Clinically relevant ARG list, analysis data, nonpareil coverage, reads per sample, MAG accession information.
