# Supplementary material for: Environmental exposures associated with the gut microbiome and resistome of pregnant women and children in Northwest Ecuador
Source: Nat Commun. 2025 Dec 13;17:15. doi: 10.1038/s41467-025-66567-1 (PMC12764814; doi:10.1038/s41467-025-66567-1)
Supplement: Supplementary file 4 — Reporting Summary [file 41467_2025_66567_MOESM4_ESM.pdf]

nature portfolio

Corresponding author(s): Levy and Fuhrmeister

Last updated by author(s): Oct 9, 2025

Reporting Summary

Nature Portfolio wishes to improve the reproducibility of the work that we publish. This form provides structure for consistency and transparency in reporting. For further information on Nature Portfolio policies, see our Editorial Policies and the Editorial Policy Checklist.

Statistics

For all statistical analyses, confirm that the following items are present in the figure legend, table legend, main text, or Methods section.

n/a	Confirmed	
☐	☒	The exact sample size (*n*) for each experimental group/condition, given as a discrete number and unit of measurement
☐	☒	A statement on whether measurements were taken from distinct samples or whether the same sample was measured repeatedly
☐	☒	The statistical test(s) used AND whether they are one- or two-sided<br>*Only common tests should be described solely by name; describe more complex techniques in the Methods section.*
☐	☒	A description of all covariates tested
☒	☐	A description of any assumptions or corrections, such as tests of normality and adjustment for multiple comparisons
☐	☒	A full description of the statistical parameters including central tendency (e.g. means) or other basic estimates (e.g. regression coefficient) AND variation (e.g. standard deviation) or associated estimates of uncertainty (e.g. confidence intervals)
☐	☒	For null hypothesis testing, the test statistic (e.g. *F*, *t*, *r*) with confidence intervals, effect sizes, degrees of freedom and *P* value noted<br>*Give P values as exact values whenever suitable.*
☒	☐	For Bayesian analysis, information on the choice of priors and Markov chain Monte Carlo settings
☒	☐	For hierarchical and complex designs, identification of the appropriate level for tests and full reporting of outcomes
☒	☐	Estimates of effect sizes (e.g. Cohen's *d*, Pearson's *r*), indicating how they were calculated

Our web collection on statistics for biologists contains articles on many of the points above.

Software and code

Policy information about availability of computer code

Data collection	We used the following open-source software for metagenome sequence analysis: bbtagger v3.101, multitrim v1.2.6., blast v2.5.0, kraken2 v2.1.3, metaSpades v3.15.5, Prodigal v2.6.3, abricate v1.0.1, PlasX v0.0.0 , MetaBAT2 v2.12.1, MaxBin2 v2.2.7, dRep v3.4.0, CheckM v1.2.2, GTDB-Tk v2.3.2, DIAMOND v2.0.15 , enveomics toolkit, MicrobeCensus v1.1.1, StrainGE v1.3.9.
Data analysis	We used the following open source software for data analysis: R v4.4.1, R's glm() , geeglm(), vif(), waldtest function in R. All codes for analysis are available on a publicly available github (https://github.com/fuhr-microlab/ecomid_amr)

For manuscripts utilizing custom algorithms or software that are central to the research but not yet described in published literature, software must be made available to editors and reviewers. We strongly encourage code deposition in a community repository (e.g. GitHub). See the Nature Portfolio guidelines for submitting code & software for further information.

Data

Policy information about availability of data

All manuscripts must include a data availability statement. This statement should provide the following information, where applicable:

- Accession codes, unique identifiers, or web links for publicly available datasets
- A description of any restrictions on data availability
- For clinical datasets or third party data, please ensure that the statement adheres to our policy

All fastq files generated in this study and metagenome-assembled genomes have been deposited in the Sequence Read Archives under BioProject PRJNA1225421. Data used for linear models are provided in the supporting information.

Research involving human participants, their data, or biological material

Policy information about studies with human participants or human data. See also policy information about sex, gender (identity/presentation), and sexual orientation and race, ethnicity and racism.

Reporting on sex and gender	Sex and gender subgroups for linear models are reported. By default, mothers were all reported female.
Reporting on race, ethnicity, or other socially relevant groupings	An asset score was created as a proxy for SES using ownership data on 15 household possessions and multiple correspondence analysis. The asset score was divided into quartiles, representing SES, with 1 indicating the lowest and 4 the highest SES.
Population characteristics	Communities were grouped into four categories with varying urbanicity: (1) Urban: the city of Esmeraldas (population ~162,000), (2) Intermediate: the town of Borbón, (population ~5,000), (3) Rural-road: rural villages that are accessible by road near Borbón, (populations ~500-1000), and (4) Rural-river: rural villages that are mostly accessible by river with some limited car accessibility, (populations ~200–700).
Recruitment	Potential participants were identified by screening women receiving late pre-natal care at public hospitals or clinics in each of the three sites: (i) local health centers in the communities in the Canton of Eloy Alfaro (rural sites), (ii) Borbón Hospital (Borbón site), and Hospital Delfina Torres de Concha (Esmeraldas site). The study team has prior relationships with all three sites, as well as with regional and national level authorities (Ecuadorian Ministry of Health). The study team worked with hospital staff to identify potential study participants, i.e., women attending prenatal care who meet the clinical inclusion and exclusion criteria. If the clinical screening criteria were met, the woman were contacted by the study team, who applied additional eligibility criteria. Families that met all inclusion criteria were invited to participate in the study via the informed consent procedure.
Ethics oversight	The study protocol was approved by the institutional review boards of the University of Washington (UW; STUDY00014270), Emory University (IRB00101202), the University of California, San Francisco (21–33932), the Universidad San Francisco de Quito (USFQ; 2018–022M), and the Ecuadorian Ministry of Health (MSPCURI000253–4).

Note that full information on the approval of the study protocol must also be provided in the manuscript.

Field-specific reporting

Please select the one below that is the best fit for your research. If you are not sure, read the appropriate sections before making your selection.

☒ Life sciences ☐ Behavioural & social sciences ☐ Ecological, evolutionary & environmental sciences

For a reference copy of the document with all sections, see nature.com/documents/nr-reporting-summary-flat.pdf

Life sciences study design

All studies must disclose on these points even when the disclosure is negative.

Sample size	Sample size was under powered for adults. 53 samples from mothers and 93 samples from children (1-week, 3-months and 6-months) were available prior to the COVID-19 pandemic project pause. To expand our analysis of environmental exposures, we selected an additional 60 stool samples from 18-month-old children. 30 of these samples were collected during the pre-COVID-19 enrollment period and corresponded to children who were already included in earlier time points (e.g., 1 week, 3 months, and 6 months). This allowed us to assess exposure over a longer developmental window (18 months). The remaining 30 samples were collected from children enrolled after the COVID-19 pause. These samples were selected to obtain a more balanced distribution of animal exposure across households.
Data exclusions	No data were excluded.
Replication	Analyses were replicated by IC and VA.
Randomization	Participants were not randomized.
Blinding	Investigators were not blinded.

Reporting for specific materials, systems and methods

We require information from authors about some types of materials, experimental systems and methods used in many studies. Here, indicate whether each material, system or method listed is relevant to your study. If you are not sure if a list item applies to your research, read the appropriate section before selecting a response.

Materials & experimental systems

n/a	Involved in the study
☒	☐ Antibodies
☒	☐ Eukaryotic cell lines
☒	☐ Palaeontology and archaeology
☒	☐ Animals and other organisms
☒	☐ Clinical data
☒	☐ Dual use research of concern
☒	☐ Plants

Methods

n/a	Involved in the study
☒	☐ ChIP-seq
☒	☐ Flow cytometry
☒	☐ MRI-based neuroimaging

Plants

Seed stocks	NA
Novel plant genotypes	NA
Authentication	NA
